# Acanthamoeba Keratitis: 34-Year Epidemiological Profile

**DOI:** 10.3390/antibiotics15050488

**Published:** 2026-05-12

**Authors:** Saad H. AlEnezi, Shaimaa Mohammed Alrefaie, Adi Mohammed Al Owaifeer, Hani Basher AlBalawi, Naif Mamdouh Alali, Mohammad Alabduljabbar, Shaker O. Alreshidi, Moustafa S. Magliyah, Entesar A. Altalhi, Shaima Sulaiman Alharbi, Abdulaziz S. Alharthi, Yousef A. Alotaibi, Saad S. Alharbi

**Affiliations:** 1 Ophthalmology Division, Department of Surgical Specialities, College of Medicine, Majmaah University, Majmaah 11952, Saudi Arabia; s.alreshidi@mu.edu.sa (S.O.A.); e.altalhi@mu.edu.sa (E.A.A.); sh.alharbi@mu.edu.sa (S.S.A.); a.alharthi@mu.edu.sa (A.S.A.); 2 King Khaled Eye Specialist Hospital, Riyadh 11462, Saudi Arabia; srefaie@kkesh.med.sa (S.M.A.); aalowaifeer@kfu.edu.sa (A.M.A.O.); mabduljabbar@kkesh.med.sa (M.A.); mmagliyah@kkesh.med.sa (M.S.M.); salharbi@tec.med.sa (S.S.A.); 3 Ophthalmology Unit, Department of Surgery, College of Medicine, King Faisal University, Al-Ahsa 31982, Saudi Arabia; 4 Division of Ophthalmology, Department of Surgery, Faculty of Medicine, University of Tabuk, Tabuk 71491, Saudi Arabia; hb.albalawi@ut.edu.sa (H.B.A.); nmalali@ut.edu.sa (N.M.A.); 5 Department of Ophthalmology, College of Medicine, King Khalid University, Abha 61421, Saudi Arabia; yalotaibi@kku.edu.sa; 6 The Eye Consultants Center, Riyadh 12333, Saudi Arabia

**Keywords:** acanthamoeba keratitis, contact lens, microbial keratitis, penetrating keratoplasty, Saudi Arabia, anti-amoebic therapy, visual outcome

## Abstract

**Background/Objectives**: *Acanthamoeba keratitis* (*AK*) is a rare but sight-threatening corneal infection. This study reviews the clinical profile, diagnostic pathways, treatment strategies, and outcomes of AK cases managed over a 34-year period. **Methods**: We conducted a retrospective analysis of 52 microbiologically AK cases from 1983 to 2017. **Results**: The mean age at presentation was 27.7 ± 9.4 years, with a female predominance (63.5%). The majority (82.7%) were contact lens users, almost exclusively soft lens wearers, with documented risk behaviors such as poor hygiene and sleeping with lenses. 44.2% were initially misdiagnosed as nonspecific microbial keratitis. Common clinical findings included epithelial defects (30.8%), ring infiltrates (44.2%), superficial infiltrates (53.8%), hypopyon (30.8%), and corneal thinning (13.5%). Diagnosis was confirmed by culture/stain in 61.5% of cases, while others required confocal microscopy or corneal biopsy. Co-infections with bacteria were noted in ~20%. Prior to referral, 82.7% of patients had received empirical topical therapy. At KKESH, all received dual anti-Acanthamoeba therapy, and 69.2% underwent surgical intervention, including tectonic PKP (46.2%) and optical PKP (19.2%). Visual acuity improved from a mean logMAR of 1.51 at presentation to 0.87 at last follow-up. Anti-Acanthamoeba therapy was discontinued in 95.9% of patients by the end of follow-up, with steroid use tapering from 61.5% at 3 months to 16.3% at final visit. **Conclusions**: Acanthamoeba keratitis in Saudi Arabia predominantly affects young female contact lens users and often presents with diagnostic delays and misclassification as herpetic or bacterial keratitis. Despite aggressive medical and surgical therapy, visual outcomes remain suboptimal in many cases.

## 1. Introduction

*Acanthamoeba keratitis* (*AK*) is a rare, sight-threatening corneal infection caused by free-living protozoa of the *Acanthamoeba* species. Though less common than bacterial or fungal keratitis, AK poses considerable diagnostic and therapeutic challenges due to its variable presentation, slow progression, and frequent resistance to standard antimicrobials. The disease is most frequently encountered among contact lens (CL) wearers and can lead to significant corneal morbidity and vision loss if not diagnosed early.

Globally, contact lens use remains the predominant risk factor. In a 15-year retrospective study from Australia, Höllhumer et al. reported that 83% of Acanthamoeba keratitis cases occurred in contact lens users [1]. The study highlighted poor lens hygiene and exposure to water sources—such as swimming pools and rinsing lenses with tap water—as significant predisposing risk factors. Similarly, in a UK-based case–control study (Carnt et al., 2018) [2], over 90% of Acanthamoeba keratitis cases were in contact lens users. The study identified key modifiable risk factors—including poor lens and hand hygiene, water exposure (e.g., swimming or bathing with lenses), and use of non-peroxide disinfection systems (such as Oxipol)—underscoring the importance of compliant lens care practices [2].

In Saudi Arabia, a small-scale report by Alfawaz A. (2011) [3] described two cases of Acanthamoeba keratitis exclusively in soft contact lens users. Both patients had exposure to key modifiable risk behaviors, including overnight wearing of lenses and rinsing lenses with tap water [3].

Diagnostic delays are common, with many patients initially misdiagnosed as having herpetic or bacterial keratitis. In their South Indian tertiary-care review (1991–2000), Gopinathan et al. found that over half of patients with microbial keratitis—including Acanthamoeba cases—had been initially treated elsewhere, often with antimicrobials, suggesting frequent early misdiagnosis as bacterial or fungal keratitis [4]. Culture negativity was common, reinforcing the need for high clinical suspicion and diagnostic adjuncts. Moreover, later studies like Vaddavalli et al., 2011 confirm that in vivo confocal microscopy is a valuable tool (88% sensitivity, 91% specificity) for diagnosing Acanthamoeba keratitis when routine methods fail [5].

An international retrospective review by Kitzmann et al. (2009) [6] of AK cases treated with therapeutic keratoplasty (1980–2007) found that over half (55%) required multiple grafts. The median postoperative visual acuity was 20/40, with a substantial proportion remaining at 20/200 or worse. These findings reinforce that despite advances in pharmacotherapy, visual outcomes in advanced or late-presenting AK cases remain guarded [6].

Given the increased prevalence of contact lens use in Saudi Arabia and the persistent diagnostic and therapeutic challenges of AK, our study aims to provide a comprehensive 34-year review of Acanthamoeba keratitis cases managed at King Khaled Eye Specialist Hospital (KKESH), a national referral center in Riyadh. By examining demographic trends, clinical features, diagnostic pathways, treatment modalities, and outcomes, this study seeks to contribute to improved awareness, earlier recognition, and more effective management of AK in the region.

## 2. Results

A total of 52 patients were diagnosed with *Acanthamoeba keratitis* (*AK*) at King Khaled Eye Specialist Hospital (KKESH) between November 1983 and December 2017. A synthesis of demographic, clinical, diagnostic, and outcome data is summarized below using structured tables and visual analytics.

### 2.1. Patient Demographics and Referral Patterns (Table 1)

Among the 52 patients diagnosed with *Acanthamoeba keratitis* (*AK*), the mean age at onset was 27.7 ± 9.4 years (range 10.5–47.8), with a female predominance (63.5%). The vast majority were Saudi nationals (96.2%), while only two were non-Saudis. Geographically, cases clustered most in the central (30.8%) and eastern (21.2%) regions, with fewer cases from the western and southern regions (13.5% each), the northern region (5.8%), and eight patients (15.4%) with unknown residency. Only 21.2% presented directly to King Khaled Eye Specialist Hospital (KKESH), while 78.8% were referred, often after an initial misdiagnosis. The most frequent incorrect referral diagnoses were nonspecific microbial keratitis (44.2%) and herpetic keratitis (11.5%), while one patient each was diagnosed as “herpetic plus Acanthamoeba” or “other.” Only 25% were referred with a correct diagnosis of AK, underscoring the persistent diagnostic challenge.

**Table 1:** Demographics and Referral Patterns (n = 52).

Characteristic	Value
Mean Age at Onset (years)	27.7 ± 9.4 (Range: 10.5–47.8)
Sex	Male: 19 (36.5%), Female: 33 (63.5%)
Nationality	Saudi: 50 (96.2%), Non-Saudi: 2 (3.8%)
Region of Residency	Central: 16 (30.8%), Eastern: 11 (21.2%), Western: 7 (13.5%), Southern: 7 (13.5%), Northern: 3 (5.8%), Unknown: 8 (15.4%)
Initial Visit Type	Presented to KKESH directly: 11 (21.2%), Referred from outside: 41 (78.8%)
Referral Diagnosis	Nonspecific microbial keratitis: 23 (44.2%), Herpetic: 6 (11.5%), Acanthamoeba: 13 (25.0%), Herpetic + Acanthamoeba: 1 (1.9%), Other: 1 (1.9%), Unclear: 8 (15.4%)

### 2.2. Clinical Background and Risk Factors (Table 2)

Most patients (80.8%) had no significant systemic comorbidities. Of the 19.2% with medical illnesses, asthma (5.8%), sickle cell anemia (3.8%), and hypothyroidism (1.9%) were documented, with a few others having combined conditions. Contact lens (CL) wear was the overwhelming risk factor, seen in 82.7% of patients, nearly all of whom (97.7%) used soft lenses. Documented high-risk behaviors, including poor hygiene, swimming, and overnight wear, were identified in 9.6%. Non-contact-lens-related risk factors were rare, with ocular trauma in 3.8%, prior ocular surgery in 5.8% (mostly previous keratoplasty), and swimming exposure in 1.9%.

**Table 2:** Systemic and Ocular Risk Factors.

Factor	Frequency (%)
No Systemic Illness	42 (80.8%)
Comorbidities (total)	10 (19.2%)
– Bronchial Asthma	3 (5.8%)
– Sickle Cell Anemia	2 (3.8%)
– Hypothyroidism	1 (1.9%)
– Others/Combinations	4 (7.7%)
Contact Lens Use	43 (82.7%)
– Soft lenses	42 (97.7% of CL users)
– Rigid lenses	1 (2.3% of CL users)
– Documented risk behaviors	5 (9.6%)
Ocular Trauma	2 (3.8%)
Swimming in Public Pools	1 (1.9%)
Previous Ocular Surgery	3 (5.8%)
– Penetrating Keratoplasty (PKP)	2 (3.8%)
– Other	1 (1.9%)

### 2.3. Clinical Presentation and Examination (Table 3)

At presentation, the right eye was slightly more commonly affected (57.7%) than the left (42.3%). The mean best-corrected visual acuity (BCVA) was poor at 1.51 ± 0.81 logMAR (equivalent to ~20/650 Snellen), while intraocular pressure averaged 16.0 ± 4.2 mmHg. Corneal findings were variable: epithelial defects were present in 30.8% of cases, ring infiltrates in 44.2%, and superficial stromal infiltrates in 53.8%. Other findings included corneal thinning (13.5%), endothelial plaques (3.8%), and nonspecific changes (30.8%). Hypopyon was observed in 30.8% of patients, while scleritis and lens status changes were rare (1.9% each). No cases of endophthalmitis were recorded. These findings illustrate the diverse and often advanced clinical spectrum of AK at presentation.

**Table 3:** Clinical Presentation and Findings.

Parameter	Value/Frequency
Affected Eye	Right: 30 (57.7%), Left: 22 (42.3%)
Mean BCVA at Presentation	1.51 ± 0.81 logMAR (Range: 0.18–3.00)
Mean IOP at Presentation	16.0 ± 4.2 mmHg (Range: 10–27)
Corneal Findings	
– Epithelial Defect	16 (30.8%)
– Ring Infiltrate	23 (44.2%)
– Superficial Infiltrates	28 (53.8%)
– Corneal Thinning	7 (13.5%)
– Endothelial Plaques	2 (3.8%)
– Others	16 (30.8%)
Associated Ocular Complications	
– Hypopyon	16 (30.8%)
– Scleritis	1 (1.9%)
– Lens Status Change	1 (1.9%)
– Endophthalmitis	0 (0%)

### 2.4. Diagnostic Work-Up (Table 4)

Culture and staining remained the cornerstone of diagnosis, confirming 61.5% of cases, while confocal microscopy and corneal biopsy contributed to 11.5% and 5.8% of diagnoses, respectively. Combined culture and biopsy were used in 15.4%, and one case was diagnosed after tectonic keratoplasty. Two patients (3.8%) were diagnosed using clinical impression or scraping. Multiple staining methods were applied across patients, most frequently Calcofluor white, Gram, and Giemsa stains (each in 15 patients, ~26%), and Gomori methenamine silver (GMS) stain in 5%. Co-infections were not uncommon: Gram-positive bacteria (76.9%) were most frequent, followed by mixed bacterial (15.4%) and rare fungal associations. Of note, most patients (80%) had no corneal scraping attempted before diagnosis, reflecting a reliance on referral-based diagnosis and highlighting potential delays in work-up.

**Table 4:** Diagnostic Modalities Used.

Diagnostic Method	No. of Patients (%)
Culture and Stain	32 (61.5%)
Confocal Microscopy	6 (11.5%)
Corneal Biopsy	3 (5.8%)
Tectonic PKP	1 (1.9%)
Culture + Biopsy (combined)	8 (15.4%)
Others (clinical, scraping)	2 (3.8%)

Note: Multiple stains were applied in several cases (Calcofluor, Gram, Giemsa, GMS). Coinfections included Gram-positive bacteria, Gram-negative, mixed, and occasional fungal organisms.

### 2.5. Medical and Surgical Management (Table 5)

The majority of patients had already received topical therapy before referral, most often biguanides (82.7%), neomycin (80.8%), or diamidines (15.4%). Chlorhexidine use was noted in 9.6%. Nearly all patients (90.4%) received topical steroids, while additional topical antivirals (13.5%) and antibiotics (73.1%) were commonly prescribed. Systemic treatments were less frequent, used in 17.3% of patients, and included antivirals (7.7%) and miscellaneous agents. Surgical intervention was required in 69.2% of cases, reflecting the severity of disease. Tectonic keratoplasty was performed in 46.2%, while optical keratoplasty was indicated in 19.2%. Other common procedures included amniotic membrane transplantation (15.4%) and cataract surgery (15.4%). Additional interventions such as glaucoma surgery, YAG capsulotomy, photorefractive keratectomy, and patch grafts were performed in select cases.

**Table 5:** Medical and Surgical Management.

Treatment Type	Frequency (%)
Topical Anti-Acanthamoeba	
– Biguanide	43 (82.7%)
– Diamidine	8 (15.4%)
– Chlorhexidine	5 (9.6%)
– Neomycin	42 (80.8%)
– Azole	38 (73.1%)
Topical Steroids	47 (90.4%)
Topical Antivirals	7 (13.5%)
Topical Antibiotics	38 (73.1%)
Systemic Therapy	
– Antibiotic	1 (1.9%)
– Antiviral	4 (7.7%)
– Azole	1 (1.9%)
– Other	3 (5.8%)
Surgical Interventions	
– Tectonic PKP	24 (46.2%)
– Optical PKP	10 (19.2%)
– Amniotic Membrane Transplant	8 (15.4%)
– Cataract Surgery	8 (15.4%)
– Superficial Keratectomy	1 (1.9%)
– Other Surgeries (glaucoma tube, YAG, PRK, patch graft, etc.)	17 (32.7%)

### 2.6. Postoperative and Longitudinal Outcomes (Table 6)

Visual outcomes improved significantly with treatment: mean BCVA improved from 1.51 logMAR at baseline to 0.87 logMAR at final follow-up, corresponding to a shift from severe visual impairment to moderate impairment. Therapeutic burden also declined over time. By three months, only 27.5% had discontinued anti-Acanthamoeba therapy, but by the last visit, nearly all (95.9%) were off therapy. Similarly, steroid use decreased from 61.5% at three months to 16.3% at the final visit, reflecting resolution of inflammation and careful tapering. Overall, these outcomes suggest that with aggressive multimodal management, including surgery in many cases, meaningful visual and clinical recovery can be achieved in this challenging infection.

**Table 6:** Postoperative and Longitudinal Outcomes.

Outcome Measure	Value
Mean BCVA improvement	From 1.51 logMAR to 0.87 logMAR
Anti-Acanthamoeba therapy discontinued by 3 months	27.5%
Anti-Acanthamoeba therapy discontinued by last visit	95.9%
Steroid use at 3 months	61.5%
Steroid use at last visit	16.3%

## 3. Discussion

Our study provides a comprehensive overview of *Acanthamoeba keratitis* (*AK*) cases at a tertiary eye center and highlights key similarities and differences with findings from the literature on national, regional, and global scales [7]. The mean age at onset in our cohort (~27.7 years) highlights that AK predominantly affects young adults in their prime contact lens-wearing years. This is in line with numerous studies worldwide. For instance, Alreshidi et al. reported that 73% of AK patients in Saudi Arabia were ≤39 years old [8]. Females comprised 63.5% of our cases, a finding echoed by other authors who noted a female preponderance (e.g., Alreshidi et al. also found 73% female in their series). This trend is often attributed to the higher usage of contact lenses among young women. Indeed, contact lens use was the dominant risk factor in our study (82.7% of patients), which mirrors the consensus in the literature that contact lens wear is the single greatest risk factor for AK. Nearly all recent AK case series from developed nations report the vast majority (often 85–95%) of patients are contact lens wearers [8,9]. For example, a nationwide Dutch survey found 95% of AK patients were contact lens users [9], and a New Zealand study noted contact lens history in 96–100% of cases [10]. By contrast, in environments where contact lens use is less common, other risk factors predominate. A striking example comes from Bharathi et al. in rural south India, who found 100% of their AK patients had a history of corneal trauma, typically soil or mud injury, with none related to contact lenses [11]. Those patients were largely young male agricultural workers, reflecting a very different epidemiological pattern. Thus, our findings of a contact lens–centric etiology align with national and global reports from urban centers, while traumatic AK remains a concern in agrarian settings.

### 3.1. Referral Patterns and Misdiagnosis

In our cohort, only 21.2% of patients presented to our center directly, whereas 78.8% were referred from elsewhere, often with an initial misdiagnosis. The referral diagnoses were frequently nonspecific (44.2%) or mistaken as herpetic keratitis (11.5%). This highlights a well-known challenge in AK management: early clinical features can mimic other corneal infections, leading to diagnostic delay. The literature strongly emphasizes this issue. Illingworth et al. reported that 77% of AK cases in the UK were initially misdiagnosed as viral keratitis, most commonly herpes simplex [12]. Similarly, McKelvie et al. noted that only 12% of AK cases in New Zealand were correctly identified at first presentation, with ~15% misdiagnosed as viral keratitis [10]. Such delays are critical–studies have shown that prolonged symptom duration before appropriate therapy is associated with worse outcomes. In the UK, Carnt et al. found that symptom duration > 5 weeks and a misdiagnosis of HSV (often leading to corticosteroid use) were independent risk factors for corneal perforation or need for transplant [2]. Our data concur, as a number of referred patients had been treated as herpetic or “microbial” keratitis with topical steroids before AK was recognized. The use of topical steroids prior to diagnosing AK is particularly detrimental. Carnt et al. (Moorfields Eye Hospital) reported that corticosteroid use before anti-amoebic therapy was associated with nearly a two-fold increase in risk of a “bad outcome” (e.g., need for keratoplasty or final vision worse than 20/80) [2]. Likewise, a Dutch study by Randag et al. identified steroid pre-treatment and indirect (delayed) referral to a cornea specialist as significant predictors of treatment failure [9]. These findings underscore the importance of early recognition of AK and caution against empirical steroid use in undiagnosed keratitis. Our study reinforces this message: misdiagnosis and delayed appropriate referral were common, suggesting a need for heightened clinical suspicion of AK, especially in young contact lens users presenting with keratitis unresponsive to standard therapy.

### 3.2. Clinical Features

The classic clinical manifestations of Acanthamoeba keratitis observed in our patients are consistent with those reported in the literature, with some variation in frequency. We found that 44.2% of cases had the ring-shaped stromal infiltrate classically associated with AK. This proportion is comparable to other studies: for example, Alreshidi et al. reported ring infiltrates in ~54% of AK cases [8], and Illingworth’s series noted rings in roughly half of patients as well (approximately 50% in their subset) [12]. Radial perineuritis (radial keratoneuritis), another hallmark often described in AK, was not explicitly quantified in our series, but it was noted qualitatively in some severe cases. Other authors have found a wide range in its occurrence—Alreshidi et al. observed radial keratoneuritis in 46% of AK eyes [8], whereas some older studies reported it in a smaller subset, likely depending on disease stage at presentation. It’s well recognized that early AK can present with nonspecific epitheliopathy or pseudodendritic lesions before classic signs develop. In our cohort, epithelial defects were present in 30.8% of patients at presentation, while superficial infiltrates were observed in 53.8%. A minority (~23%) had an atypical “pseudodendritic” or superficial infiltrate pattern noted in their charts, which can be confused with herpes simplex infection. We also documented a relatively high rate of hypopyon (30.8%) and anterior chamber reaction in our patients. While Acanthamoeba keratitis is typically limited to the corneal stroma, a subset of severe or late-presenting cases exhibit intense inflammation. For example, an international tertiary-care review [13] found hypopyon in 4.6% of eyes—most of which had large ulcers—while a recent Indian review similarly noted hypopyon, uveitis, and stromal ulceration as hallmark features of advanced AK [14]. Scleritis was very rare in our series (1.9%), consistent with the literature that scleritis is an uncommon but grave complication of AK. Importantly, pain out of proportion to clinical signs was a frequent symptom in our patients (not captured in our tables but noted anecdotally), aligning with the well-known clinical clue that AK causes disproportionate ocular pain due to perineural inflammation. Acanthamoeba keratitis often causes severe, disproportionate ocular pain due to intense perineural inflammation. Perineuritis—observed in 2.5–63% of cases—likely results from trophozoite-driven protease activity around corneal nerves, explaining why patients report significant pain even with relatively mild corneal findings [15].

Overall, our clinical findings support those of prior reports: a combination of ring infiltrate, epithelial defects, and (when present) radial keratoneuritis should raise strong suspicion for AK [8], even if any one sign is absent. Our experience also reiterates that AK can masquerade as more benign entities early on, which again underscores the need for careful evaluation of contact lens wearers with keratitis.

### 3.3. Diagnosis

In our study, definitive diagnosis of Acanthamoeba was achieved by culture or cytology in 61.5% of cases, reflecting the reliance on microbiological confirmation. We used non-nutrient agar cultures with *E. coli* overlay and various stains (Calcofluor white, Giemsa, Gram, etc.) to identify trophozoites/cysts. This success rate is on par with what is reported in other tertiary centers. A significant consideration in interpreting these findings is the process in which diagnostic approaches have changed over time. Earlier than 2000, diagnosis was based primarily on culture and light microscopy, which are limited sensitivity and turnaround time, with reported culture positivity rates ranging from 0% to 53% [16,17]. In contrast, more recent decades have seen increasing adoption of in vivo confocal microscopy, improved staining techniques, and clinical awareness, all of which have significantly better early detection and diagnostic accuracy.

However, we also note that in vivo confocal microscopy was utilized in only 11.5% of our patients, a figure lower than in some reports. Confocal microscopy has become an invaluable tool in many centers for rapid, non-invasive diagnosis. McKelvie et al. reported confocal confirmation in 67% of AK cases in Auckland, with an estimated sensitivity of ~83% [10]. In vivo confocal microscopy has become a cornerstone for diagnosing Acanthamoeba keratitis. Kobayashi et al. (2013) [18] and Winchester et al. (1995) [19] visualized characteristic 10–20 µm reflective cysts directly in the corneal epithelium and stroma. A more recent Manchester-based series (Przybek Skrzypecka et al., 2025) [20] reported an 85% detection of AK via IVCM, frequently identifying double-walled cysts, bright spots, signet-ring structures, and even trophozoites. The more limited use of confocal in our series may be due to resource and timing constraints; many patients had already undergone corneal scraping at referring hospitals or had deep stromal involvement less amenable to confocal visualization. We did find an important subset (15.4%) who required multiple diagnostic modalities (culture plus biopsy) to finally confirm the diagnosis, highlighting that a high index of suspicion must be maintained and invasive diagnostic procedures repeated if initial tests are negative and clinical suspicion remains high. Our diagnostic yield for Acanthamoeba improved when multiple methods were employed, consistent with global recommendations. For example, Li et al. from China advocate using Giemsa-stained smears, saline wet mounts and confocal in suspected early AK, noting that each modality can catch cases the others miss [21]. In resource-limited settings, simpler techniques have proven their worth: the 10% KOH wet mount, for instance, showed higher sensitivity for cyst detection in an Indian study [11], and many laboratories in our region use Calcofluor white staining (which binds to cyst walls) as a quick diagnostic aid. Another notable finding in our cohort was the occurrence of microbial co-infections. We identified concomitant bacteria in ~20% of culture-positive AK cases (mostly Gram-positive cocci, and one case of fungal co-infection). Coinfections are documented elsewhere—Alreshidi et al. also reported a few mixed infections (Acanthamoeba with bacteria or fungus) in their series [8]. Clinically, co-infections with bacteria, fungi, or viruses have been reported in up to 23% of AK cases, complicating diagnosis and contributing to atypical presentations and misdiagnosis (Carnt et al., 2025) [19].

These can confound the clinical picture and potentially explain why some of our patients were initially treated solely for bacterial keratitis. The message is that a multimodal diagnostic approach is often necessary for AK: combining clinical suspicion with smears, cultures, and confocal microscopy improves detection, as evidenced in both our experience and the literature.

### 3.4. Treatment

The management practices and outcomes in our study broadly align with standard care and outcomes reported in other studies, though with a few distinctions. Earlier management often involved delayed initiation of anti-amoebic therapy and inappropriate corticosteroid use prior to diagnosis. Contemporary approaches emphasize early initiation of biguanides (PHMB or chlorhexidine), combination therapy with diamidines, and cautious, delayed use of corticosteroids after infection control is achieved (Carnt et al., 2025) [19]. The recognition of polymicrobial keratitis also has therapeutic implications, as mixed infections may require broader antimicrobial coverage and may partially explain suboptimal response to monotherapy in some cases.

All of our patients received aggressive topical anti-Acanthamoeba therapy, most commonly a dual regimen of biguanides (polyhexamethylene biguanide 0.02% or chlorhexidine 0.02%) and diamidines (propamidine isethionate Brolene drops). This regimen is considered first-line and was also used in the vast majority of cases in UK series like Illingworth et al. (where 100% received PHMB and propamidine) [12]. Additionally, 80.8% of our patients received adjunctive topical neomycin sulfate (often as part of combination drops or to cover potential bacterial co-infection), and 73.1% received a topical azole (voriconazole or ketoconazole), reflecting a broad-spectrum approach especially early in the course when initial diagnoses were uncertain. One controversial area is the use of topical corticosteroids in AK. In our cohort, 90.4% of patients were eventually treated with topical steroids as the infection came under control, to reduce inflammation and corneal scarring. We typically introduce steroids in a delayed fashion—several weeks into therapy—once there are signs that trophozoites are being eliminated. This approach finds support in some literature, as controlled steroid use can improve pain and limit immune-mediated damage; however, it must be done cautiously. Our high rate of steroid use likely also reflects that many referrals were already on steroids (for presumed other diagnoses) before AK was confirmed. The pros and cons of steroids in AK are debated: a retrospective analysis by Carnt et al. [2] suggested that steroid use prior to anti-amoebic treatment was associated with worse outcomes, but steroid use after appropriate therapy began did not significantly affect the odds of needing surgery. Another study by Robaei et al. (2014) [22] evaluated adjunctive topical corticosteroid therapy initiated after the start of anti-amoebic treatment in Acanthamoeba keratitis. They found that, in eyes without hypopyon or scleritis at baseline, judicious steroid use did not worsen final visual outcomes—despite sometimes prolonging time to cure—supporting the safety of controlled steroid addition once infection control is underway [22].

Our practice aligns with a carefully timed use of steroids, and the fact that 16.3% of patients were still on topical steroids at last follow-up (typically for graft rejection prophylaxis or chronic inflammation) highlights the long-term management needed in severe AK cases.

Despite intensive medical therapy, surgical intervention was frequently required in our series. Almost half of the patients (46.2%) underwent at least one therapeutic penetrating keratoplasty (PKP) to remove infected tissue or to manage impeding perforation. An additional 19.2% received an optical PKP later for visual rehabilitation. This combined keratoplasty rate (~65% receiving some form of corneal transplant) is high, albeit comparable to other reports from tertiary centers dealing with advanced AK. Across tertiary-care centers, approximately 20–35% of Acanthamoeba keratitis cases require surgical intervention—including therapeutic keratoplasty—often due to delayed presentation or severe disease. For instance, a German series reported PKP in 22.7% of cases [13], while another cohort found that 35.5% of patients underwent surgery, and UK data show poor outcomes (including surgery) in nearly half of patients [2]. Our higher rate may reflect our role as a referral center receiving very severe cases (often after weeks of delayed treatment). It is known that the likelihood of needing keratoplasty increases with diagnostic delay: Illingworth et al. observed that all eyes in their series that needed PKP had at least a 6-week delay in diagnosis [12]. In our study, many of the 24 tectonic PKPs were performed within the first 2–3 months of presentation due to advanced corneal melting or perforation risk, suggesting those patients had significant delays before reaching us. Encouragingly, some recent studies report lower surgical rates with earlier intervention; for instance, McKelvie et al. in New Zealand reported only 4 out of 52 patients (7.7%) needed transplant, and those were cases with the longest diagnostic delays (2–4 months) [10]. This further underlines the value of early diagnosis. Apart from keratoplasty, we also employed other surgeries: 15.4% required amniotic membrane transplantation to promote epithelial healing, 15.4% eventually needed cataract extraction (likely steroid-induced cataracts in many cases), and one case required a conjunctival flap. A small number of patients needed glaucoma drainage devices (5.8%) due to secondary glaucoma from prolonged steroid use or uveitis. These interventions underscore that AK management can be prolonged and complex, often involving multiple subspecialties.

The presence of polymicrobial infections also has therapeutic implications. Mixed infections may require combined anti-amoebic and antibacterial/antifungal therapy. Failure to identify co-infection may lead to poor or delayed response.

### 3.5. Outcomes

The visual outcomes in our cohort illustrate both the improvements achievable with treatment and the fact that many patients are left with substantial visual impairment. The mean best-corrected visual acuity (BCVA) improved from logMAR 1.51 (~20/640 Snellen) at presentation to logMAR 0.87 (~20/150) at final follow-up. In practical terms, this is an improvement from legal blindness range to a level of vision that, while much better, is still far from normal. Only a minority of our patients recovered near-normal vision (for example, final VA 20/40 or better was achieved in relatively few cases, often those who had early disease or successful optical PKP). This outcome profile aligns with many contemporary reports of AK, especially from referral centers. Randag et al. found that despite aggressive management, about 39% of AK patients in the Netherlands suffered “treatment failure,” defined as needing surgical intervention or ending with poor vision [9]. Carnt et al. similarly reported almost half (48%) of their 194-patient cohort had a bad outcome by composite criteria (which included final VA ≤ 20/80) [2]. These figures mirror our finding that a substantial proportion of patients have lasting vision loss. On the other hand, if AK is caught early, outcomes can be dramatically better—Illingworth et al. (1995) reported that 78% of eyes achieved 20/20 vision after treatment, attributing this success to prompt diagnosis and treatment before the infection caused deep stromal damage [12]. Our study unfortunately had many late presenters, which likely explains why our visual results skew modest. It is worth noting that among eyes that did not require therapeutic keratoplasty in our series, the outcomes were generally better (several achieved 20/40–20/25 with only medical therapy and possibly later optical PKP). This dichotomy is well-recognized: eyes with milder AK or early treatment often do well, whereas those with severe AK (especially if compounded by delays or steroid misuse) can end up needing transplants and may still have guarded vision.

We also documented the lengthy treatment duration characteristic of AK. At 3 months into follow-up, 72.5% of patients were still on active anti-amoebic therapy, and 50% at 6 months. A quarter remained on treatment at 1 year, and a few (≈5%) continued beyond 2 years for chronic, recalcitrant infection. This protracted course is commonly noted in the literature. For example, Moorfields data indicated a median therapy duration of around 6–9 months, with the most severe quartile requiring ≥10–12 months of treatment [2]. AK’s cyst form can persist and cause relapse if therapy is prematurely halted, hence the need for prolonged treatment and careful tapering. Our use of topical anti-amoebics was gradually reduced over many months, parallel to what is recommended globally. By the last follow-up, only 4.1% of patients remained on anti-Acanthamoeba drops, implying that the vast majority did eventually achieve clinical resolution of infection, albeit some with significant scarring.

### 3.6. Comparison with Regional and Global Trends

Regionally, our findings concur with reports from the Middle East that contact lens misuse is driving an increase in AK cases. In Iran and neighboring countries, studies have observed similar demographics and risk profiles. Niyyati et al., for instance, found that 30% of keratitis cases in their series were due to Acanthamoeba, all of which occurred in soft contact lens users (with a female predominance of ~87%) [23]. This is very much in line with our Saudi data and highlights that the Middle East’s growing adoption of cosmetic and corrective contact lenses has introduced Western-like AK epidemiology in the region. The Middle East literature also contains reminders that non-lens-related AK does occur: for example, a case report from Turkey described an AK in a child with a history of swimming in contaminated water and playing with soil (genotype T9 in that case) [24], and a Saudi case report by Alfawaz et al. documented AK associated with traditional eye medicine use in a non-lens wearer [3]. These outliers notwithstanding, the consensus regionally is that the vast majority of AK cases are lens-related today, and our study reinforces that. Nationally, AK has been considered relatively rare in Saudi Arabia, with earlier reviews noting a lower incidence compared to Western countries. Our series (52 cases over 34 years in a single center) and the recent report by Alreshidi et al. (24 cases over 5 years at the same hospital) [8] suggest that while AK remains less common than bacterial or fungal keratitis, it is now regularly encountered. Public health measures in Saudi Arabia–such as high-quality water treatment–may reduce environmental exposure to Acanthamoeba [25], but the risk from improper contact lens handling remains a critical concern. Alarmingly, global trends show AK incidence is rising. A New Zealand study documented a doubling of AK cases in the 2010s compared to the prior decade [10], and the Dutch nationwide survey recorded an increase from 16 cases in 2009 to 49 cases in 2015 (estimated incidence ~1 in 21,000 contact lens wearers per year by 2015) [9]. Similar increases have been noted in the UK and USA, often following outbreaks related to contact lens solutions or water supply issues. These data place our findings in context: the challenge of Acanthamoeba keratitis is growing, and our region is part of this global trend.

## 4. Materials and Methods

We conducted a retrospective chart review of all patients diagnosed with AK at KKESH from November 1983 through December 2017, following institutional review board approval. All demographic and clinical data were extracted. Predisposing factors (e.g., CL wear, trauma, water exposure) and initial misdiagnoses were noted. The presenting features were categorized by stage: mild stage (epithelial defects, pseudo-dendritic lesions, punctate epitheliopathy, perineural infiltrates, superficial stromal infiltration) versus advanced stage (deep stromal infiltrates, ring infiltrates, endothelial plaques, corneal thinning/perforation, hypopyon, or scleritis). Microbiological work-up followed standard protocol: corneal scrapings were stained (H&E, Gram, Giemsa) and cultured on blood, chocolate, Sabouraud, and thioglycollate media. In suspected AK cases, additional culture was done on non-nutrient agar with an *Escherichia coli* overlay. If smears were negative but clinical suspicion remained high, diagnosis was confirmed by in vivo confocal microscopy or corneal biopsy. Corneal scraping is the collection of corneal material for direct smear examination and culture. Staining (H&E, Gram, Giemsa) and culture are the following steps in laboratory processing of these specimens.

Treatment regimens were divided into standard therapy and adjuvant therapy. Standard therapy consisted of anti-amoebic agents given topically: either monotherapy (one drug) or dual therapy (two drugs). Monotherapy agents included propamidine isethionate 0.1%, polyhexamethylene biguanide (PHMB) 0.02%, or chlorhexidine 0.02%. Dual therapy used propamidine combined with PHMB or chlorhexidine. Adjuvant therapy (added for refractory cases) included additional topical/oral agents such as voriconazole, neomycin, or oral itraconazole. Initial therapy was started with monotherapy or dual therapy; if the response was poor, an adjuvant agent was added. Medical treatment continued for several weeks after resolution of active keratitis and inflammation. Therapeutic penetrating keratoplasty (TPK) was performed in cases of perforation or unresponsive infection; optical keratoplasty was reserved for inactive disease (minimum 3 months quiescent) to improve vision.

Treatment failure was defined as final best-corrected visual acuity (BCVA) worse than 20/100 and/or needing corneal transplantation (therapeutic or optical). Based on this definition, outcomes were classified as “healed” or “failed,” and we analyzed factors associated with failure. Statistical comparisons (e.g., between mild vs. advanced stage) were done using appropriate tests (*t*-test, chi-square) with *p* < 0.05 considered significant [7].

## 5. Conclusions

In summary, our study’s findings of young, female contact lens wearers being predominantly affected, frequent initial misdiagnosis (especially as herpetic keratitis), and the necessity for prolonged dual anti-amoebic therapy (often supplemented by cautious steroid use) are well corroborated by national, regional, and international studies. We report substantial rates of surgical intervention and only modest visual recovery in many patients, outcomes that mirror those from other tertiary care centers managing advanced AK. The comparison across studies emphasizes several key points for improving care: the need for greater contact lens hygiene education (as poor lens habits were noted in our series and others), the importance of early diagnosis and referral (to avoid the delays that worsen prognosis), and continued research into better diagnostic tools and treatments (given that current medical therapy, though effective at eradicating infection, often does not fully restore vision). Our findings contribute to the growing body of evidence that Acanthamoeba keratitis is a significant public health ophthalmic issue–one that transcends borders–and underscore the urgency of preventive strategies and early intervention to preserve vision in at-risk patients.

## Data Availability

All the data presented in this manuscript are available on request.

## Abbreviation

The following abbreviation is used in this manuscript:

*AK*	*Acanthamoeba keratitis*
